# A Novel Pectic Polysaccharide of Jujube Pomace: Structural Analysis and Intracellular Antioxidant Activities

**DOI:** 10.3390/antiox9020127

**Published:** 2020-02-02

**Authors:** Ximeng Lin, Keshan Liu, Sheng Yin, Yimin Qin, Peili Shen, Qiang Peng

**Affiliations:** 1 College of Food Science and Engineering, Northwest A&F University, Yangling 712100, China; ximenglin423@nwsuaf.edu.cn (X.L.); Liukeshan@nwafu.edu.cn (K.L.); 2 Beijing Engineering and Technology Research Center of Food Additives, Beijing Technology & Business University (BTBU), Beijing 100048, China; yinsheng@btbu.edu.cn; 3 State Key Laboratory of Bioactive Seaweed Substances, Ministry of Agriculture Key Laboratory of Seaweed Fertilizers, Qingdao Brightmoon Seaweed Group Co Ltd., Qingdao 266400, China; yiminqin1965@126.com

**Keywords:** polysaccharide, jujube pomace, structural analysis, antioxidant activity

## Abstract

After extraction from jujube pomace and purification by two columns (DEAE-Sepharose Fast Flow and Sepharcyl S-300), the structure of SAZMP4 was investigated by HPGPC, GC, FI-IR, GC-MS, NMR, SEM, and AFM. Analysis determined that SAZMP4 (Mw = 28.94 kDa) was a pectic polysaccharide mainly containing 1,4-linked GalA (93.48%) with side chains of 1,2,4-linked Rha and 1,3,5-linked Ara and terminals of 1-linked Rha and 1-linked Ara, which might be the homogalacturonan (HG) type with side chains of the RG-I type, corresponding to the results of NMR. In AFM and SEM images, self-assembly and aggregation of SAZMP4 were respectively observed indicating its structural features. The antioxidant activity of SAZMP4 against H_2_O_2_-induced oxidative stress in Caco-2 cells was determined by activity of superoxide dismutase (SOD) and glutathione peroxidase (GSH-Px) as well as malondialdehyde (MDA) and reactive oxygen species (ROS) levels, indicating SAZMP4 can be a natural antioxidant. Also, a better water retention capacity and thermal stability of SAZMP4 was observed based on DSC analysis, which could be applied in food industry as an additive.

## 1. Introduction

Pectin is a natural macromolecular compound, generally considered as a complex polysaccharide containing α-1,4-linked galacturonic acid, which might be partly methyl esterified and have side chains of various neutral sugars, such as rhamnose, arabinose, galactose, and so on [1]. It widely exists in the cell wall and the middle lamella structure of all higher plants [2]. Because of pectin’s gelatification, thickening, and stabilization, it is widely applied in food, medical, chemical, and other industries [3]. China, the original country of jujube, has cultivated jujube since around 7000 years ago. Since jujube fruits are rich in sugar, and the abundant intracellular and cell wall polysaccharides are more soluble in alkaline solution, it is better to use alkaline solution for extraction to take full advantage of the pomace [4]. According to previous research, jujube polysaccharides have different biological activities, such as antioxidant activity [5], immunoregulatory activity [6], hepatoprotective effects [7], anti-hyperlipidemia effects [8], and antitumor activity [9]. Obviously, biological activities of polysaccharides are associated with their structural characteristics. Many researchers have determined the composition, the average molecular weight, and the type of linkages of polysaccharides can affect the biological activities of polysaccharides [10,11,12]. Thus, there exists an importance to determine the structural characterizations of pectic polysaccharide.

It is widely acknowledged that free radicals are indispensable in metabolic processes. However, oxidative stress is an important factor in these diseases. Reactive oxygen species (ROS), chemically active substances mainly including peroxide, superoxide, hydroxyl radicals, and singlet oxygen [13], plays a necessary role in physiological regulation and message passing in moderate amount. Normally, there exists an antioxidant system containing antioxidants and antioxidant enzymes that controls the metabolic balance of free radicals. However, as a result of some unnormal factors and conditions like ischemia, hypoxia, chemicals, ionizing radiation, chemotherapy drugs, and ultraviolet radiation, abundant free radicals will be produced and break the metabolic balance. Under this circumstance, oxidative stress will occur. Also, excessive free radical accumulation can injure the components of cells, such as DNA and proteins, leading to the development and progression of diseases such as diabetes, cancer, and cardiovascular diseases [14,15]. Thus, it is imperative to improve antioxidant activity in order to prevent and control these diseases.

In this study, the structure of a pectic polysaccharide extracted from jujube pomace was characterized by chemical and instrumental methods, and the antioxidant activity was investigated by the Caco-2 cells model.

## 2. Materials and Methods

### 2.1. Materials and Reagents

The dry fruits (*Ziziphus jujuba cv.* Muzao) were provided by the Loess Plateau Experimental Orchard from Yulin in Shaanxi province, China. The chromatographic columns (DEAE-Sepharose Fast Flow and Sepharcyl S-300) were purchased from GE Healthcare Life Sciences (Piscataway, NJ, USA). The standards arabinose, fucose, galactose, glucose, mannose, rhamnose, and xylose were from Solarbio Life Sciences Co. (Beijing, China). The standards glucuronic acid and galacturonic acid were purchased from Aladdin Biochemical Technology Co., (Shanghai, China). All other chemicals were analytical grade.

### 2.2. Extraction and Purification of SAZMP4

Jujube pomace was obtained by removing the water-insoluble polysaccharide jujube powder. Alkaline extraction (0.1 M NaOH, 25 °C, 1 h) was applied to jujube pomace for obtaining crude polysaccharide. Then, an anion-exchange column of DEAE-Sepharose Fast Flow (2.6 × 100 cm), using 0.3 M NaCl as mobile phase at room temperature with a flow rate of 1.2 mL/min, and a gel-permeation chromatography column of Sepharcyl S-300 (2.6 × 100 cm), using ultrapure water as mobile phase at room temperature with a flow rate of 0.8 mL/min, were used to purify the crude polysaccharide in order to derive the purified polysaccharide, SAZMP4 [16]. 

### 2.3. Structural analysis of SAZMP4

#### 2.3.1. Physical and Chemical Analysis

The phenol-sulfuric acid method [17], the Bradford method [18], and the Folin–Ciocalteu reagent method [19] were used to measure the content of total sugar, protein, and total phenolics.

The UV-Vis spectrum was recorded by a UV7 spectrophotometer (METTLER, TOLEDO, Zurich, Switzerland) in the 200–400 nm region to detect the protein and nuclear acids [20], and the FI-IR spectrum was recorded with a Fourier transform infrared spectrometer (FI-IR, Vetex70, Bruker Co., Ettlingen, Germany) in the 4000–400 cm^−1^ region by KBr pellets to determine the primary functional groups [21].

High-performance gel-permeation chromatography (HPGPC) equipped with an Agilent 1200 series high-performance liquid chromatography system, a Waters 2414 refractive index detector, and a TSK gel G5000PWXL column (300 × 7.8 mm, Tosoh, Japan) were used to determine the homogeneity and average molecular weight of the purified polysaccharide [22]. The retention time was used to calculate the average molecular weight of SAZMP4.

Gas chromatography (GC, GC-2014, Shimadzu Co., Kyoto, Japan) with a capillary column of DB-17 (30 m × 0.25 mm × 0.25 μm, Agilent, Santa Clara, CA, US) was used to indicate the monosaccharide composition of SAZMP4 [23], and the mixed standard monosaccharides were used for the monosaccharide identification and quantification.

A differential scanning calorimeter (DSC, Q2000, Waters, Milford, MA, USA) was used to analyze the thermal properties of SAZMP4 [24]. The dried and powdered polysaccharide (3 mg) was put into a standard aluminum crucible and sealed immediately. The program raised the temperature from 40 °C to 300 °C at a rate of 10 °C/min in a dynamic inert nitrogen atmosphere (50 mL/min). Simultaneously, an empty standard aluminum crucible was used as a reference.

#### 2.3.2. Methylation Analysis

After the uronic acid reduction by Taylor and Conrad [25], SAZMP4 was methylated by a method reported previously [26]. The disappearance of the absorption band of O-H around 3400 cm^−1^ in the FI-IR spectrum indicated complete methylation of the sample. Then, the sample was hydrolyzed by trifluoroacetic acid, restored with sodium borohydride, acetylated by acetic anhydride, and dissolved in chloroform. A GCMS-QP2010A instrument (Shimadzu Co., Kyoto, Japan) equipped with a Rtx-50 capillary column (30 m × 0.25 mm × 0.25 μm) and an ion trap MS detector was used to determine the derivatives.

#### 2.3.3. NMR Analysis

The dried SAZMP4 was dissolved (D_2_O) and lyophilized three times. Fifty milligrams of deuterium-exchanged SAZMP4 was dissolved in 0.5 mL D_2_O. NMR spectra of ^1^H and ^13^C were recorded with a Brucker AVANCE Ⅲ 500 MHz nuclear magnetic resonance spectrometer (NMR) using standard pulse sequences at 25 °C [27].

### 2.4. Molecular Morphological Analysis

A field emission scanning electron microscope (SEM, S-4800, Hitachi, Tokyo, Japan) was used to record the surface morphological properties of SAZMP4 [28]. Before observation, SAZMP4 was covered with a gold layer.

An atomic force microscope (AFM, Multimode-8, Bruker Co., Billerica, MA, USA) was used to document the properties of the molecular morphology of the polysaccharide. Ten microliters of the polysaccharide solution (1 μg/mL) was dropped onto a mica carrier and then dried at room temperature, using tapping mode on the AFM for record [29].

### 2.5. Antioxidant Activity of SAZMP4

#### 2.5.1. Cell Culture

Human colorectal adenocarcinoma cells (Caco-2) were obtained from Shanghai Institute of Cell Biology (Shanghai, China). The cells were cultured in high-glucose Dulbecco’s modified Eagle’s medium (H-DEME, Hyclone, Logan, UT, USA) with 10% fetal bovine serum (FBS, Biological Industries Beit Haemek, Kibbutz, Israel), 100 units/mL penicillin, and 100 μg/mL streptomycin and in a humidified atmosphere of 5% CO_2_ at 37 ℃. Between 3 and 15 passages of the cells were used in this study. 

#### 2.5.2. Cell Viability Analysis

Cell Counting Kit-8 (CCK-8, EnoGene Co., Shanghai, China) was used to evaluate the cell viability. In brief, Caco-2 cells were cultured in 96-well plates with a density of 5 × 10^3^ cells/mL and incubated for 24 h in a 37 °C incubator with a humidified 5% CO_2_ atmosphere. After that, the cells were treated with different concentrations of SAZMP4 (50, 100, 200, 400, and 800 μg/mL) for 24 h. Then, 10 μL of CCK-8 solution was added, and the cells were incubated in the same environment for 1 h. The cell viability was determined by a multifunctional enzyme marker (victorX3, PerkinElmer Co., Waltham, Massachusetts, US) at a wavelength of 450 nm and was expressed as a relative percentage to the blank control group.

#### 2.5.3. Treatment Procedure

For treatment, the cells (5 × 10^3^ cells/mL) were cultured in 96-well plates and incubated at 37 °C for 24 h. Then, the cells were treated with different concentrates of SAZMP4 (25, 50, 100, and 200 μg/mL) for 24 h. After removing the medium, the cells were exposed to 200 μM of H_2_O_2_ for 2 h. The cell viability, superoxide dismutase (SOD), glutathione peroxidase (GSH-Px), ROS, and malondialdehyde (MDA) levels were determined by relevant commercial kits.

#### 2.5.4. Measurement of SOD and GSH-Px

Cell lysates treated without or with different concentrations of SAZMP4 were collected for antioxidant enzymes (SOD and GSH-Px) analysis. The activities of SOD were measured by the relevant commercial kits (Beyotime, Biotechnology, Shanghai, China) using the xanthine oxidase method for determination. The activity of SOD was defined as the corresponding SOD content when the SOD inhabitation rate in each milliliter of reaction liquid reached 50%.

The activities of GSH-Px were measured by the relative commercial kits (Beyotime, Biotechnology, Shanghai, China). The activities of GSH-Px were determined by the consumption of GSH in enzymatic reactions.

#### 2.5.5. Intracellular ROS and MDA Levels

The intracellular ROS was determined by a Reactive Oxygen Species Assay Kit (Beyotime, Biotechnology, Shanghai, China), investigated by fluorophore 2,7-dichlorofluorescein diacetate (DCFH-DA). After incubating the cells in a black 96-well plate for 24 h and removing the medium, the cells were washed with phosphate-buffered saline (PBS, 100 μL). After that, the cells were cultured with DCFH-DA at a concentration of 10 μM at 37 °C for 30 min. The results were determined by a multimode microplate reader (PerkinElmer, Waltham, MA, USA) and expressed as fold changes in fluorescence intensity versus control.

The MDA levels were indicated by corresponding detection kits (Jiancheng Bioengineering Institute, Nanjing, China) following the manufacturer’s instructions.

### 2.6. Data Analysis

All test data were expressed as mean ± SD from no fewer than three determinations and analyzed with variance (ANOVA) following multiple tests. SPSS version 22.0 was used for all statistical analyses, and *p* < 0.05 was considered to be significant.

## 3. Results

### 3.1. Separation and Purification of SAZMP4

SAZMP4 was extracted from jujube pomace by alkaline solution and purified by the column of DEAE-Sepharose Fast Flow with mobile phase of 0.3 M NaCl and the column of Sepharcyl S-300 with ultrapure water. The yield of crude polysaccharide was 5.3% relative to jujube pomace, and the yield of SAZMP4 was 5.10% relative to crude polysaccharide. Other jujube researchers [5,7,9] obtained similar results to this study.

### 3.2. Preliminary Characterizations of SAZMP4

According to the phenol-sulfuric acid assay, SAZMP4 was 96.52% sugar. It had a low protein content of 0.78%, coinciding with UV–Vis analysis that the polysaccharide contained no protein (<3%) based on the no absorption peaks at 280 nm. Also, no absorption peaks at 260 nm in the spectrum indicated no nucleic acid in SAZMP4. The total phenol content was not detected in SAZMP4. These results were similar to other acidic jujube polysaccharides from *Z. Jujuba* [30].

**Figure 1:**
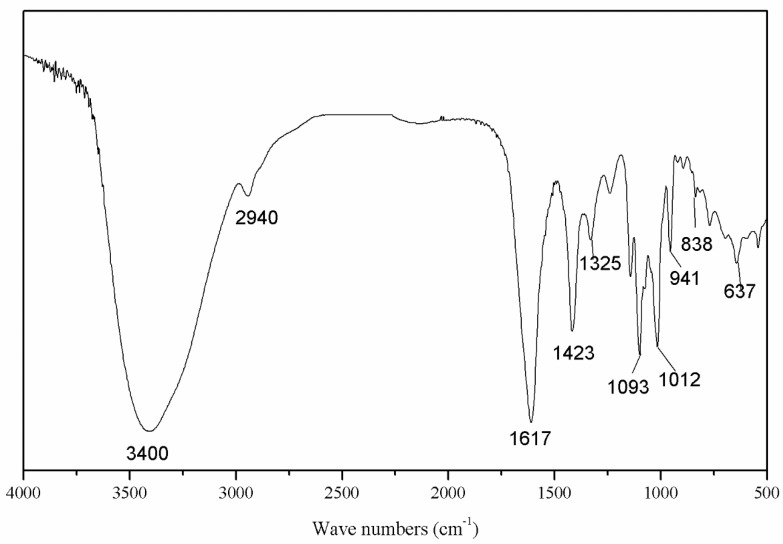
FI-IR spectrum of SAZMP4.

In the FI-IR spectrum (Figure 1), the peaks of the intramolecular or intermolecular stretching vibration of O-H was around 3400 cm^−1^ and the stretching vibration of C–H was around 2940 cm^−1^, indicating the SAZMP4 was a polysaccharide. The peaks at approximately 1620 cm^−1^ were attributed to the stretching vibration of carboxyl, which implied that SAZMP4 might contain uronic acid [31]. The absorptions at 1420 and 1325 cm^−1^ belonged to the bending vibration of C–H. Also, the signals at 1200–800 cm^−1^ of the fingerprint area of carbohydrates indicated that the bands at 1093 and 1012 cm^−1^ were the bending vibration of C–O in the pyranose form. In addition, the weak absorption bands at 941 and 838 cm^−1^ were probably attributed to α-glycosidic bonds, further supported by the out-of-plane bending vibration of C–H at around 630 cm^−1^, which implied the presence of α-glycosidic bonds in SAZMP4 [32].

According to the equation, the average molecular weight of SAZMP4 was calculated to be 28.94 kDa with the retention time of 19.91 min. Also, the single and symmetric elution peak from the HPGPC indicated SAZMP4 was a homogeneous fraction.

GC analysis, as shown in Table 1, determined that the monosaccharide composition of SAZMP4 mainly contained galacturonic acid at a molar rate of 93.48%, which coincided with the feature of pectin. This result was similar with other pectic polysaccharides [30,33,34], but the galacturonic acid content of SAZMP4 was higher than them.

**Figure 2:**
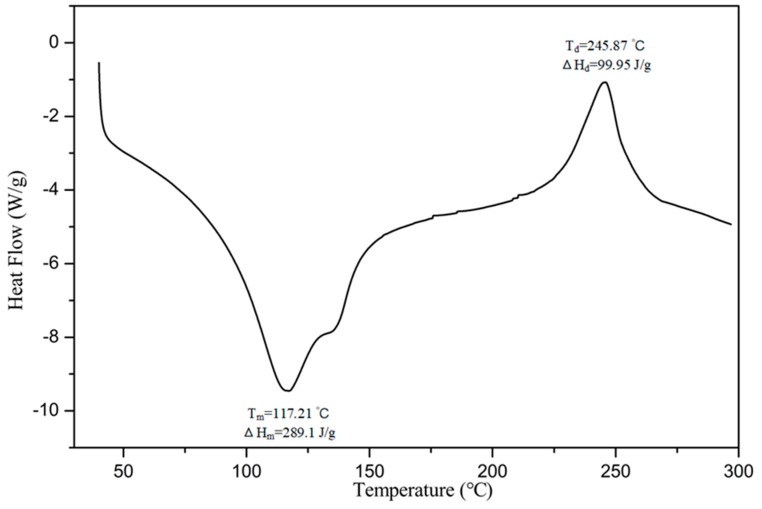
DSC thermogram of SAZMP4.

The thermodynamic properties of SAZMP4 were examined by DSC from 40 to 300 °C. In the DSC thermogram of SAZMP4 (Figure 2), an endothermic peak and an exothermic peak were observed, and the parameters of them were labeled, such as melting temperature (T_m_), melting enthalpy (ΔH_m_), degradation temperature (T_d_), and degradation enthalpy (ΔH_d_). T_m_ and ΔH_m_ were determined by the composition and structural characterizations of polysaccharides. A polysaccharide with lower molecular weight and less uronic acid content has worse capacity to sustain water, so the T_m_ and ΔH_m_ were lower [35]. The high T_m_ and ΔH_m_ indicated the better capacity of SAZMP4 to sustain water, coinciding with results of HPGPC and GC analyses. The second peak was caused by the degradation of the polysaccharide in the process [36]. Apparently, T_d_ was primarily impacted by the composition of the polysaccharides, while the ΔH_d_ of polysaccharides was mainly affected by its galacturonic acid content. The T_d_ implied that SAZMP4 was stable below 240 °C, related to the better thermal stability, which might be applied in the food industry.

### 3.3. Methylation Analysis

For the determination of linkage types between sugar residues with GC-MS, SAZMP4 was firstly subjected to uronic acid reduction in order to avoid β-condensation reaction during methylation, which might cause structural changes to sugar chains [37]. According to the retention time of sugar residues and the standard data of CCRC Spectral Database, the results of the methylation analysis are exhibited in Table 2. SAZMP4 mainly included five types of glycosidic linkages: 1-linked Rha*p*, 1-linked Ara*f*, 1,2,4-liked Rha*p*, 1,3,5-linked Ara*f,* and 1,4-linked Gal*p* with a molar ratio of 0.4: 0.38: 0.62: 0.58: 28.7. This conformed to the GC analysis, which indicated that the structures of sugar chains were not destroyed in the methylation process. Besides, GC analysis showed SAZMP4 contained only galacturonic acid, and no galactose exited in it. Thus, the linkage type of galacturonic acid was 1,4-linked GalA*p*. Obviously, SAZMP4 was a pectic polysaccharide containing 1,4-linked galacturonic acid with side chains of 1,2,4-liked Rha*p* and 1,3,5-linked Ara*f* as well as terminals of 1-linked Rha*p* and 1-linked Ara*f,* which indicated that SAZMP4 might be homogalacturonan (HG) with side chains of rhamnogalacturonan (RG) type Ι [38].

### 3.4. NMR Analysis

**Figure 3:**
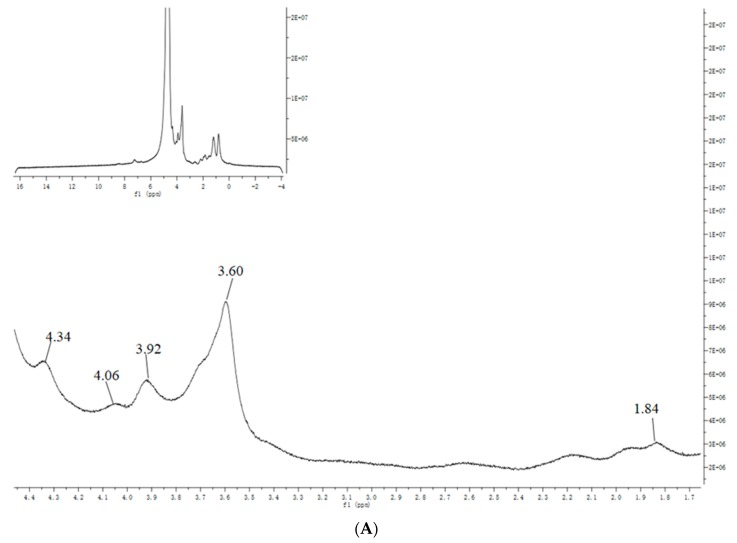

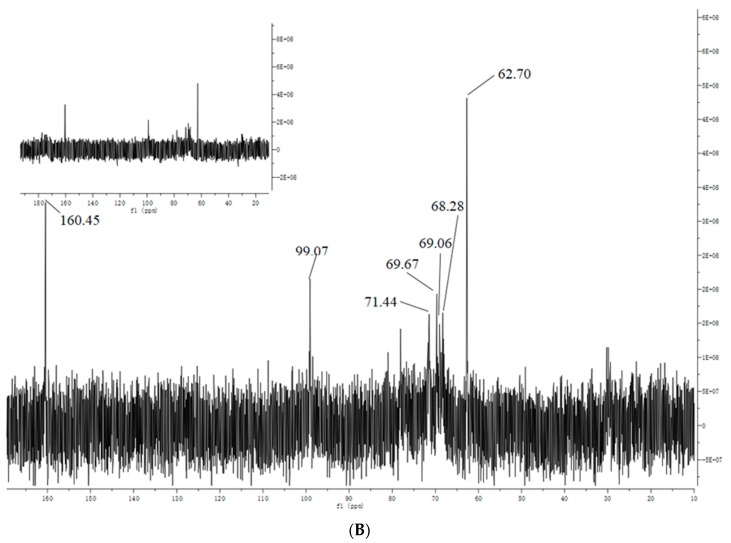
The NMR spectra of SAZMP4 in D_2_O: ^1^H spectrum (**A**); ^13^C spectrum (**B**).

The ^1^H (Figure 3A) and ^13^C NMR (Figure 3B) spectra of SAZMP4 were displayed, and the chemical shifts in the spectra were classified based on previous research [39]. In the ^1^H spectrum, the weak peak of δ 1.84 belonged to H-6 of 1,2,4-linked Rhap or 1-linked Rhap, and the other strong signs of δ 4.34, 4.06, 3.92, and 3.60 were respectively attributed to H-5, H-4, H-3, and H-2 of 1,4-linked GalAp. The strong signs of δ 160.45, 99.07, 71.44, 69.67, 68.28, and 62.70 belonged to the C-6, C-1, C-4, C-5, C-3, and C-2 of 1,4-linked GalAp in the ^13^C spectrum, respectively. 

### 3.5. Molecular Morphological Properties

**Figure 4:**
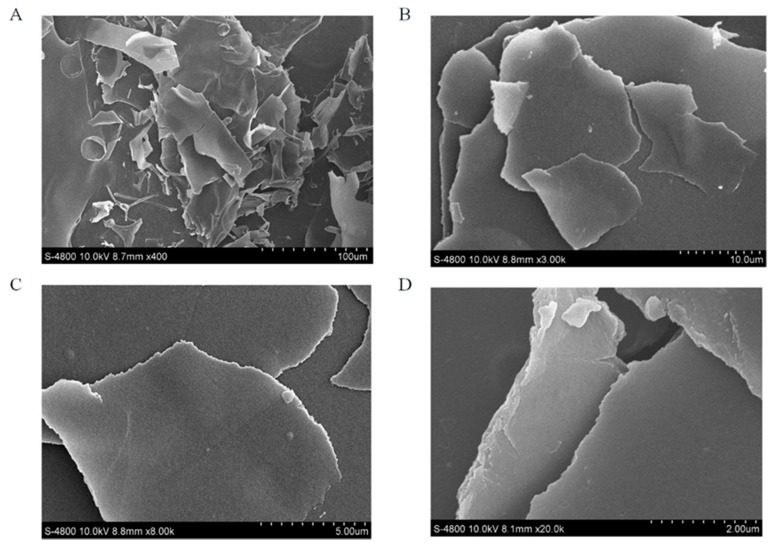
The SEM images of SAZMP4 (**A**: × 400, **B**: × 3000, **C**: × 8000, **D**: × 20,000).

It is widely acknowledged that SEM can be used to observe the surface morphology of polysaccharides, and the SEM images can exhibit the molecular morphological properties of polysaccharide. At low magnification (400-fold and 3000-fold, Figure 4A,B), SAZMP4 was observed to have a smooth surface and debris shape, while it showed a smooth surface and a thin but large lamellar shape in the image of high magnification (8000-fold and 20,000-fold, Figure 4C,D). These results were different to those of previous research [40] possibly because of the different preparation and purification methods and the structural differences of polysaccharides.

**Figure 5:**
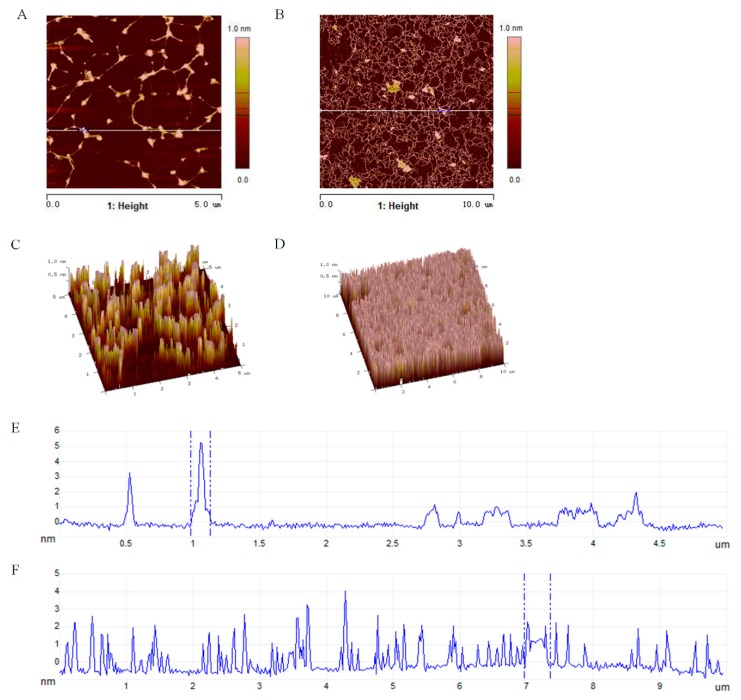
The AFM planar image (**A**) and (**B**); three-dimensional image of AFM (**C**) and (**D**); the height analysis of the planar image at the line (**E**) and (**F**).

AFM is another tool that can not only provide two-dimensional images but also observe three-dimensional surface images of polysaccharides directly in the natural conditions. Generally speaking, sugar chains with different compositions always have the tendency to form into a conformation with the lowest free energy. According to the AFM images (Figure 5B), SAZMP4 was observed to have an irregular bulk structure, indicating the molecular aggregation caused by intermolecular and intramolecular interactions of hydroxyl groups on polysaccharide chains, and these results coincided with the results of SEM analysis. The height analysis in the planar image (Figure 5E,F) revealed the aggregation extent of SAZMP4. A branched, ring-like, helical or an interconnected network structure in the AFM image (Figure 5A,B) implied molecular self-assembly of SAZMP4, which undergoes a spontaneous process from disorder to order based on the weak, noncovalent interaction of hydrogen bonds and van der Waals forces as well as the hydrophobic effect [41]. These results indicated that SAZMP4 might aggregate at first and then self-assemble to form a long chain; this is why the molecular morphology of SAZMP4 appeared the way it did in SEM and AFM images. In addition, SAZMP4 was also observed to have a core structure with branches probably related to the α-1,4 glycosidic bonds, which might be galacturonic acid, coinciding with the methylation analysis [42].

### 3.6. Antioxidant Activity of SAZMP4

#### 3.6.1. The Cytotoxicity of SAZMP4 to Caco-2 Cells

**Figure 6:**
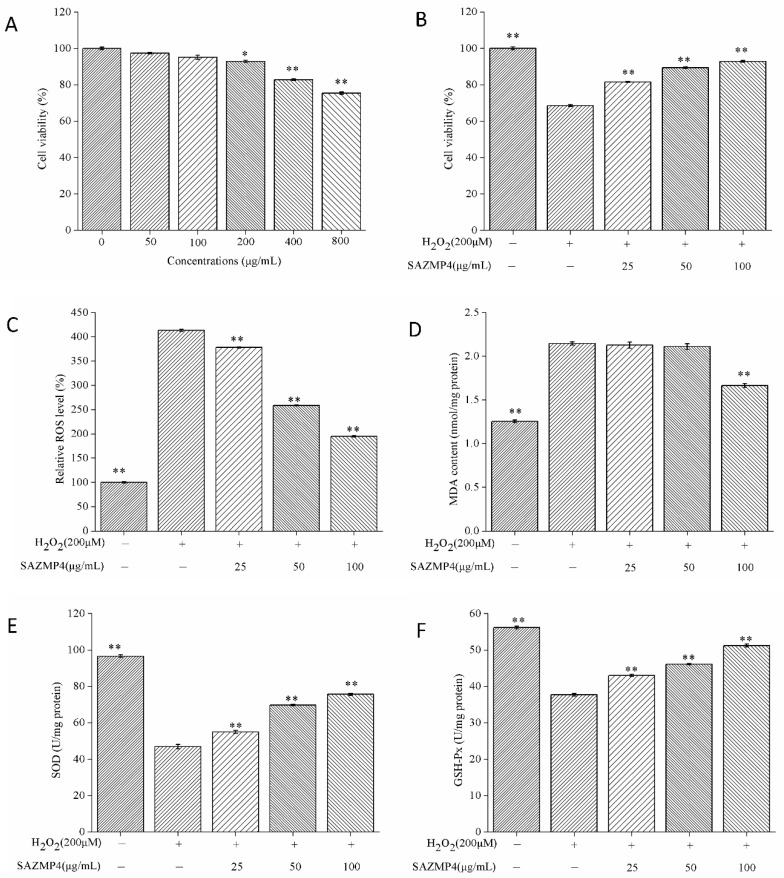
Cell viability of Caco-2 with different concentrations of SAZMP4 (**A**). Cell viability of Caco-2 with 200 μM H_2_O_2_ and different concentrations of SAZMP4 (**B**). Intracellular level of ROS in Caco-2 cells (**C**). Intracellular level of MDA in Caco-2 cells (**D**). Activity of SOD in Caco-2 cells (**E**). Activity of GSH-Px in Caco-2 cells (**F**). The data are expressed as the mean ± SD (*n* = 5 wells per group). (*) *p <* 0.05 and (**) *p <* 0.01 versus the control group.

The cytotoxic effects of different concentrations of SAMZP4 on Caco-2 cells were evaluated by the CCK-8 commercial kit. As shown in Figure 6A, SAZMP4 of 50 and 100 μg/mL presented no significant effects on cell viability compared to the control group, while SAZMP4 at high concentrations (200, 400, and 800 μg/mL) caused a significant dose-dependent decrease of cell viability. Hence, the following experiments were carried out with 25, 50, and 100 μg/mL of SAZMP4 for treatment in order to reduce the interference with the polysaccharide.

#### 3.6.2. Protective Effect against H_2_O_2_-induced Toxicity

After treating with different concentrations of SAZMP4 for 24 h, the cells were exposed to 200 μM H_2_O_2_ for 2 h. The results are exhibited in Figure 6B. The cell viability of the group only exposed to H_2_O_2_ declined to 68.6%, indicating the cells were in the state of oxidative stress. Pretreatment of SAZMP4 for 24 h enabled the cells to resist the toxic effects of H_2_O_2_, causing the viability to be greater than 81.6%. However, these results were not precise to indicate SAZMP4 had antioxidant activity. Thus, the determinations of ROS and MDA levels and SOD and GSH-Px activities were inevitable.

#### 3.6.3. ROS and MDA Levels in Caco-2 Cells

The ROS and MDA levels in Caco-2 cells after pretreatment of SAZMP4 and treatment of 200 μM H_2_O_2_ are exhibited in Figure 6C. There was a significant increase in the ROS level in the group without pretreatment of SAZMP4 compared to the control group. Pretreated groups had significant decreases of ROS levels and exhibited a dose-dependent response. Also, similar results were exhibited in the changes of MDA levels (Figure 6D). At 100 μg/mL, SAZMP4 presented significant decreases compared to the group only exposed to 200 μM H_2_O_2_. Apparently, these results suggested that SAZMP4 could help the cells resist the toxicity of H_2_O_2_.

#### 3.6.4. The Activity of SOD and GSH-Px in Caco-2 Cells

Figure 6E,F, respectively, showed the activity of SOD and GSH-Px in Caco-2 cells. Compared to the control group, the activity of SOD and GSH-Px significantly declined in the no pretreatment group. With pretreatment of SAZMP4, the activity of SOD and GSH-Px had a significant increase comparing to the 200 μM H_2_O_2_-treated group and showed a dose-dependent relationship. Obviously, these results indicated that SAZMP4 might protect the cells from oxidant injury of H_2_O_2_ by activating the antioxidant enzymes (SOD and GSH-Px). 

## 4. Discussion

Mitochondria, bearing the responsibilities of the generation of cell energy (adenosine triphosphate, ATP), the main source of ROS, and the apoptosis of cells, is the core and hub of the entire cell and its vital activities. Also, it can participate in cell signaling. All these physiological functions of mitochondria are mainly needed to regulate energy metabolism and ROS production. In normal cells, there exists a balance between oxidation and antioxidation. However, metabolic disorders of ROS in mitochondria cause oxidative stress, leading to cell apoptosis and some diseases such as cancer, cardiovascular diseases (hypertension, diabetes), and neurodegenerative diseases (Parkinson’s disease). One of the ways to cause oxidative stress is lack of antioxidants. When cells are in the state of oxidative stress, they cannot scavenge the free radicals generated by mitochondria. As previous research reported [43,44,45,46,47], the antioxidant activity of polysaccharides is associated with their composition of sugar chains, branched chains, molecular weight, substituents, and conformation. The higher the molecular weight and the higher the content of uronic acid the polysaccharide has, the stronger its antioxidant ability. In this study, the structural analysis of SAZMP4 determined that it mainly contained galacturonic acid with higher molecular weight and branched chains. Hence, SAZMP4 could become a natural antioxidant because it could be the electronic acceptor and scavenge free radicals.

As polysaccharides can only be digested in the intestinal tract, this study used Caco-2 as the cell model and H_2_O_2_ as the irritant to induce cellular oxidative stress [47]. The antioxidant system in the human body has the ability to recover and regulate itself. When the body is in a state of oxidative stress, the relevant antioxidant system will produce corresponding antioxidants to control the injury of oxidative stress. SOD is an important kind of enzyme in the antioxidant system that can catalyze superoxide anions to translate into H_2_O_2_. GSH-Px is another kind of enzyme in the human body. It can scavenge H_2_O_2_ and block the lipid peroxidation radical chain reaction to protect cell membranes and other biological tissues from oxidant injury. Compared to the blank group, the cell viability of the model group had an obvious reduction indicating the success of this oxidative stress model. The stronger activity of antioxidant enzymes (SOD and GSH-Px) and the reduction of MDA and ROS levels compared to the model group indicated SAZMP4 had antioxidant effects and could improve the antioxidant ability of the cells. However, the antioxidant mechanism of polysaccharides has not clearly determined yet. The signaling pathway of Nrf2-keap2-ARE is the primary antioxidant signaling pathway in the body [48]. Nrf2 is the most important transcription factor in this signaling pathway. Active Nrf2 dissociates with keap1 and enters the nucleus to interact with antioxidant response elements (AREs) to start the transcription of antioxidant enzyme genes. At present, many antioxidants from natural plants have been determined to play an antioxidant role by promoting the dissociation of keap1-Nrf2 and activation of Nrf2, such as curcumin and phenyl ethyl caffeic acid [49]. Since polysaccharides are macromolecules, they cannot get into the membrane. Thus, it is possible that polysaccharides are decomposed into small molecules by intestinal flora at first and then enter the cells to active Nrf2. Moreover, antioxidant polysaccharides also can serve as electronic acceptors by their hydroxyl and carboxyl groups and scavenge free radicals by their special structure to play an antioxidant role in the body. Hence, polysaccharides with more uronic acid and a higher molecular weight can have a stronger antioxidant activity.

In recent years, because of the toxicity and carcinogenesis of synthetic antioxidants, people are more interested in natural antioxidants. Thus, SAZMP4 could be used as a natural antioxidant for food and medicine industries to produce some products for the prevention and control of diseases. In this study, we only investigated the structure and intercellular antioxidant activity of SAZMP4. There is no denying that further research in mice or humans is needed in order to determine the structure–activity relationship based on this study.

## 5. Conclusions

In conclusion, SAZMP4 (Mw = 28.94 kDa) is a novel pectic polysaccharide, mainly containing 1,4-linked GalA with side chains of 1,2,4-linked Rha and 1,3,5-linked Ara and terminals of 1-linked Rha and 1-linked Ara, and it has a tendency to aggregate and self-assemble. SAZMP4 can be a natural antioxidant and can be applied in the medicine industry. In addition, SAZMP4 has a better water retention capacity and thermal stability, indicating the potential capacity to be used as an additive in the food industry. This is a systematic work investigating the structure and antioxidant activity of a novel pectic polysaccharide, and it can provide a theoretical basis in further research.

## Figures and Tables

**Table 1:** Monosaccharide analysis data of SAZMP4.

Peak No.	Retention Time (min)	Monosaccharide	Molar Radio
1	11.645	Rhamnose	1
2	12.289	Arabinose	0.9
3	12.921	Xylose	0.05
4	22.800	Mannose	0.07
5	38.020	Galacturonic acid	28.9

**Table 2:** Methylation analysis data of SAZMP4.

Peak No.	Retention Time (min)	Methylated Sugars	Linkage Patterns	Molar Radio
1	20.355	2,3,4-Me_3_-Rha*p*	1-linked Rha*p*	0.5
2	26.563	2,3,5-Me_3_-Ara*f*	1-linked Ara*f*	0.47
3	31.915	2-Me-Ara*f*	1,3,5-linked Ara*f*	0.46
4	34.707	3-Me-Rha*p*	1,2,4-linked Rha*p*	0.52
5	35.092	2,3,6-Me_3_-Gal*p*	1,4-linked Gal*p*	28.8

## Abbreviations

SAZMP4: purified polysaccharide from crude polysaccharide with sodium hydroxide solution (0.3 M); HPGPC, high-performance gel permeation chromatography; GC, gas chromatography; FI-IR, Fourier transform infrared spectroscopy; NMR, nuclear magnetic resonance; SEM, scanning electron microscopy; AFM, atomic force microscopy; DSC, differential scanning calorimeter; RG, rhamnogalacturonan; SOD, superoxide dismutase; GSH-Px, glutathione peroxidase; MDA, malondialdehyde; ROS, reactive oxygen species.
